# A retrospective study of remodeling changes of the temporomandibular joint structures in patients with unilateral posterior tooth loss

**DOI:** 10.1186/s12903-025-07594-8

**Published:** 2026-01-08

**Authors:** Yan Lv, Ching-I Huang, Yajing Fu, Wenzhe Zhang, Rui Pu, Menghan Zhang, Mengjie Wu

**Affiliations:** Stomatology Hospital, School of Stomatology, Zhejiang University School of Medicine, Zhejiang Provincial Clinical Research Center for Oral Diseases, Key Laboratory of Oral Biomedical Research of Zhejiang Province, Cancer Center of Zhejiang University, Engineering Research Center of Oral Biomaterials and Devices of Zhejiang Province, Hangzhou, 310000 China

**Keywords:** Temporomandibular joint (TMJ), Unilateral posterior tooth loss (UPTL), Three-dimensional analysis (3D), Duration of tooth loss, First molar, Unilateral chewing habit (UCH)

## Abstract

**Background:**

This study aimed to enhance understanding of how unilateral posterior tooth loss (UPTL) affects temporomandibular joint (TMJ) structures and to explore potential influencing factors on joint morphology.

**Methods:**

The study included 68 subjects (34 patients with UPTL and 34 with complete dentition), divided into the missing group (*n* = 34 joint sides), the contralateral group (*n* = 34 joint sides), and the control group (*n* = 68 joint sides). Morphological measurement of the condyle, glenoid fossa and joint spaces were performed using cone-beam computed tomography (CBCT) and Mimics software. Analyses and comparisons were made among the three groups, with subgroup analyses based on the duration of tooth loss (< 1 year and ≥ 1 year), first molar loss, and the presence of unilateral chewing habit (UCH).

**Results:**

Some significant differences among the control, the missing, and the contralateral groups were observed in measurements of the glenoid fossa and joint spaces (*P* < 0.05). When tooth loss lasted less than 1 year, the contralateral side exhibited significantly larger posterior joint spaces than the missing side (*P* < 0.05), whereas no differences were observed at ≥ 1 year. In the first molar loss subgroup, the anteroposterior and mediolateral diameters of the glenoid fossa were significantly larger on the contralateral side than on the missing side (*P* < 0.05). Moreover, unilateral chewers with UPTL exhibited significantly smaller anteroposterior condylar diameters on the preferred chewing side (*P* < 0.01).

**Conclusion:**

An overall enlargement of both the glenoid fossa and joint spaces was observed in patients following UPTL; the glenoid fossa was found to be more susceptible to bone resorption than the condyle. Factors such as the duration of tooth loss, the absence of the first molar, and UCH contribute variably to these structural changes, indicating that different tooth-loss conditions can impose distinct biomechanical impacts on the TMJ.

**Supplementary Information:**

The online version contains supplementary material available at 10.1186/s12903-025-07594-8.

## Background

The temporomandibular joint (TMJ) comprises the mandibular condyle, the glenoid fossa, and an interposed articular disc. Such a complex and intricate structure ensures an even distribution of masticatory forces during activities such as chewing, speaking, and swallowing, and also underlies the joint’s capacity for adaptive remodeling [1]. Within this dynamic framework, dental occlusion plays a crucial role in regulating mandibular position and transmitting mechanical forces to TMJ.

Intact posterior dentition is essential for effective mastication, maintaining a stable occlusal vertical dimension, and preserving proper mandibular position [2]. Tooth loss may reduce occlusal support and disrupt the balanced distribution of masticatory forces, resulting in occlusal irregularities, diminished masticatory efficiency, and altered force transmission [3].

Numerous studies have explored the association between tooth loss and TMJ structures or disorders, but have yielded conflicting evidence. Extensive research [4–6] have primarily focused on condylar bony changes following tooth loss, while some [7, 8] have investigated alterations in joint space and articular eminence inclination (AEI). Scholars [2, 4] have suggested that, compared to tooth loss alone, the number of missing teeth and the number of quadrants with missing teeth had a more potential impact on the TMJ. Some studies [7, 9] even included completely edentulous patients. Previous studies [5, 10, 11] have often employed two-dimensional (2D) imaging techniques, such as panoramic tomograms or cephalometric films, with certain limitations in measurement accuracy.

Therefore, the present study aimed to systematically characterize TMJ structural alterations associated with unilateral posterior tooth loss (UPTL) using advanced cone-beam computed tomography (CBCT). Specifically, differences in condylar dimensions, glenoid fossa size and morphology, and joint space dimensions were compared between individuals with UPTL and those with complete dentition. The potential impacts of the duration of tooth loss, the absence of the first molar, and the presence of unilateral chewing habit (UCH) on TMJ structures were also explored. Collectively, these analyses provide a more comprehensive understanding of how various factors related to UPTL contribute to TMJ remodeling.

## Methods

### Sample selection and group classification

**Fig. 1:**
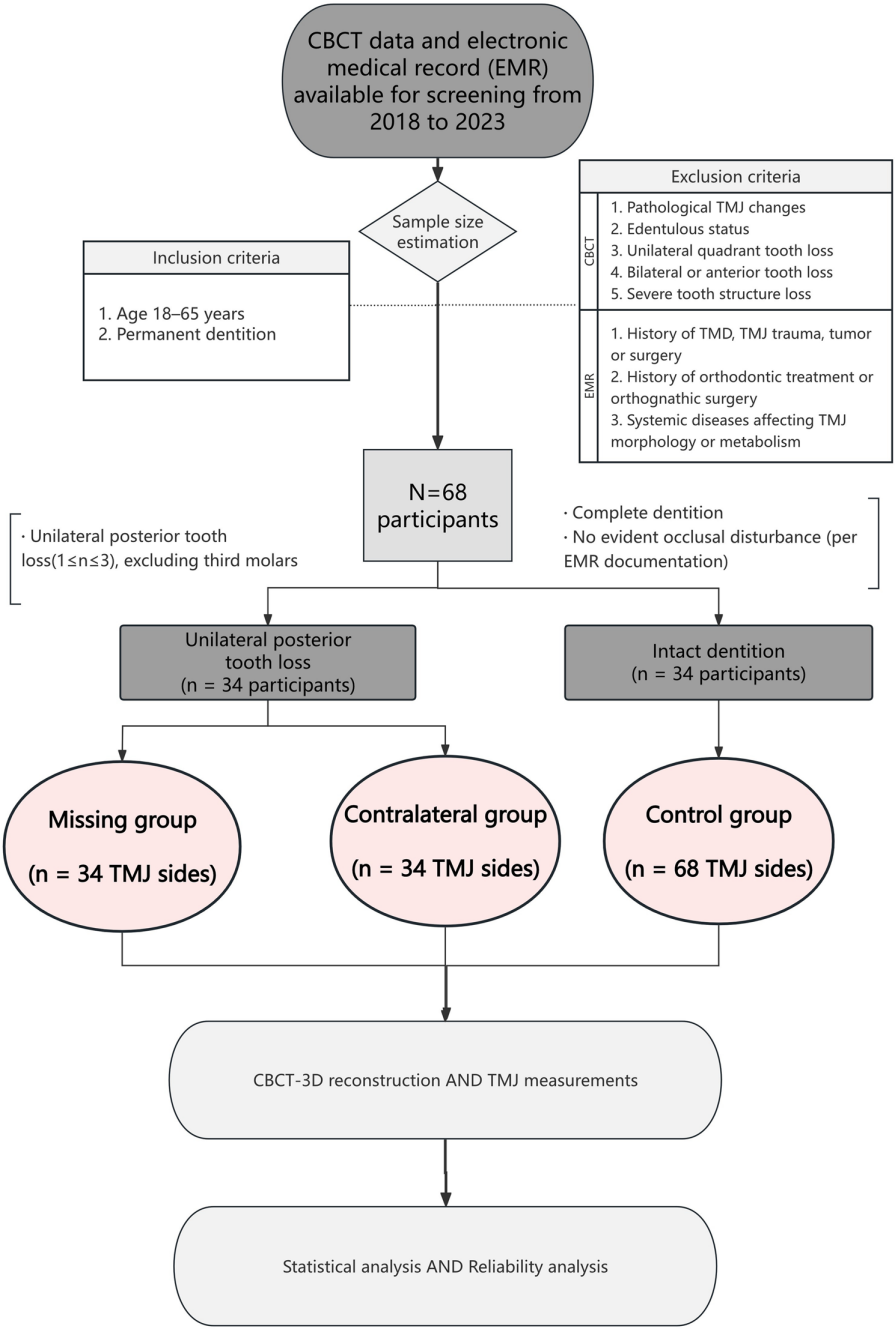
Flowchart of sample selection, group classification, 3D measurements and statistical analysis

The retrospective study reviewed CBCT images of patients who had previously undergone CBCT examinations at the Department of Implantology and the Department of Orthodontics of the Affiliated Hospital of Stomatology, School of Stomatology, Zhejiang University School of Medicine from January 2018 to December 2023 (Fig. 1). Based on archived CBCT data and electronic medical records (EMR), and according to the exclusion criteria described below, a total of 68 permanent-dentition patients aged 18–65 years (23 males and 45 females) were included.

#### Exclusion criteria based on CBCT data

1. Pathological TMJ changes;
2. Edentulous status;
3. Unilateral quadrant tooth loss;
4. Bilateral or anterior tooth loss;
5. Severe tooth structure loss.

#### Exclusion criteria based on EMR

6. History of temporomandibular disorders (TMD), TMJ trauma, tumor, or surgery;
7. History of orthodontic treatment or orthognathic surgery;
8. Systemic diseases affecting TMJ morphology or metabolism (e.g., rheumatoid arthritis).

Among the included subjects, 34 had unilateral posterior tooth loss (UPTL), defined as the loss of 1–3 posterior teeth (excluding third molars). The remaining 34 subjects had intact dentition and no unilateral chewing habit (UCH) or other evident occlusal disturbance.

TMJs were analyzed based on joint count. For patients with UPTL, the TMJ on the missing side was classified as the missing group (*n* = 34), and the contralateral side as the contralateral group (*n* = 34). For subjects with intact dentition, both TMJs were assigned to the control group (*n* = 68).

The study protocol was approved by the institutional ethics committee (Approval No. 2023-006), and all participants provided written informed consent for the use of anonymized data.

### Measurement methods and the measured items

All CBCT images were acquired by the New Tom VGI device (Quantitative Radiology, Verona, Italy) with the following parameters: 110kVp, 2.0 mA, 15*15 cm field of view, 0.3 mm voxel size and 40 s scanning time. Subjects were scanned in maximum intercuspation with the Frankfort-Horizontal (FH) plane parallel to the floor. Data were stored in Digital Imaging and Communications in Medicine (DICOM) format and imported into Mimics Medical 21.0 for 3D reconstruction. The condyle and glenoid fossa were segmented using the “Split Mask” function, converted to Stereolithography (STL) format and refined with the “Wrap” and “Smooth” tools to ensure accurate TMJ morphology.

**Fig. 2:**
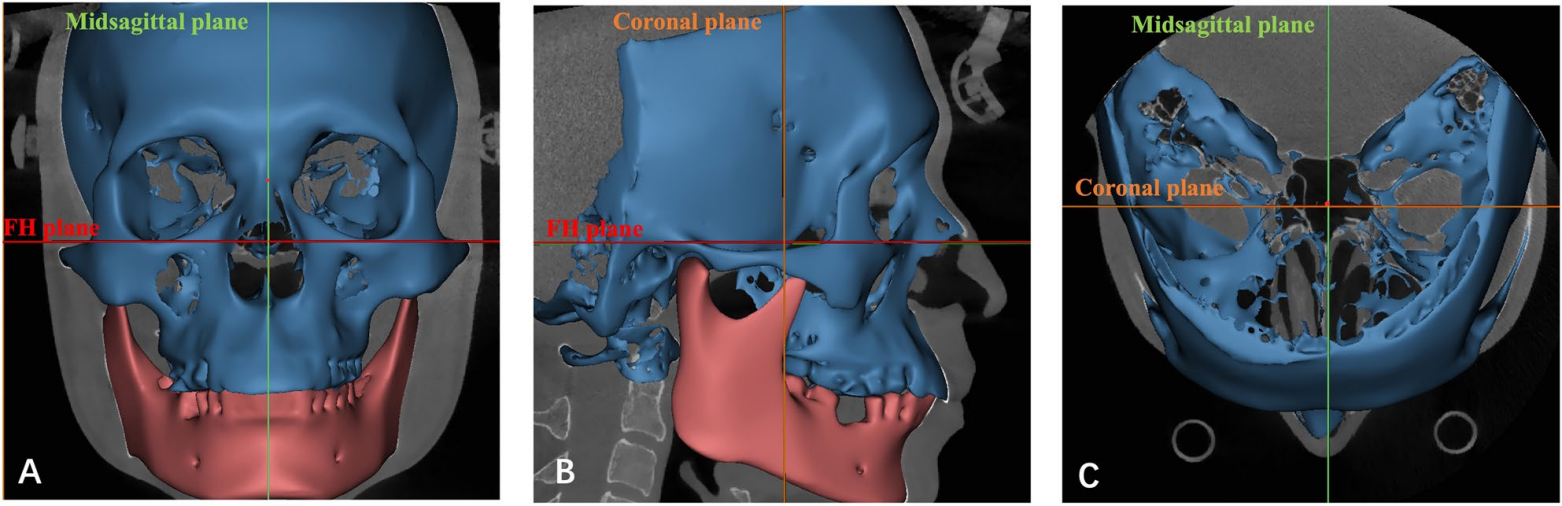
The adjusted 3D reconstructed model. **A** frontal view from the coronal plane; **B** lateral view from the midsagittal plane; **C** bottom view from the axial plane

**Fig. 3:**
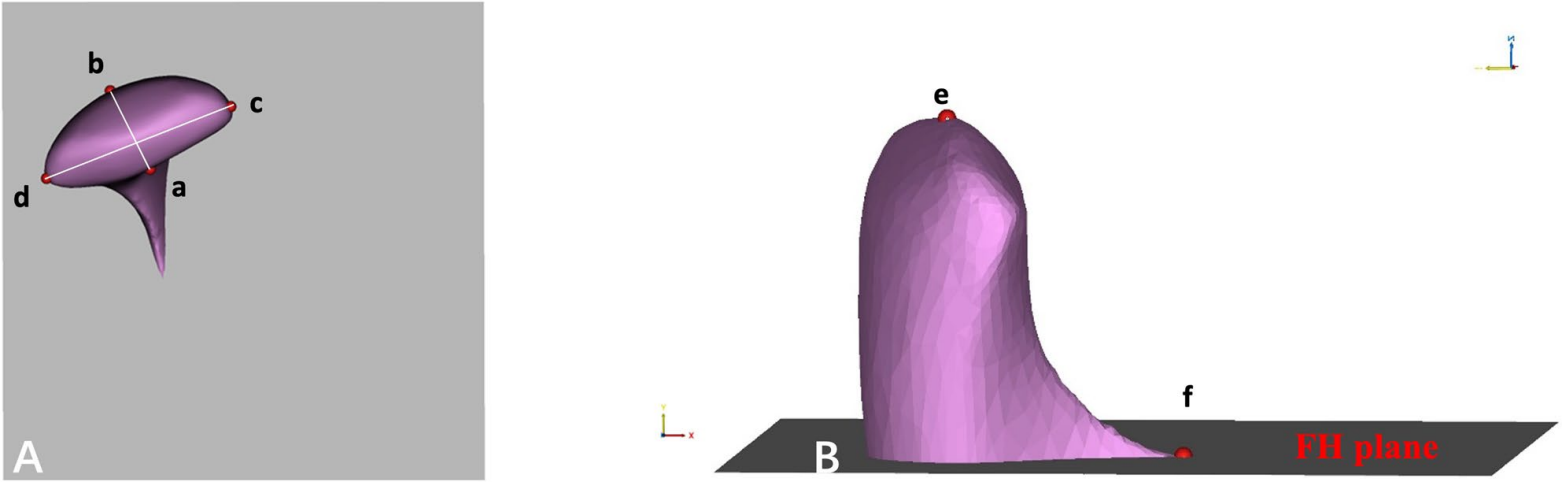
Measurements of the condyle. **A** line ab - the distance between the anterior and posterior points of the condyle, line cd - the distance between the medial and lateral points of the condyle; **B** the area and volume calculated by sectioning the mandibular 3D model from the plane parallel to the FH plane and passing through the sigmoid notch point, point e - the highest point on the condylar head, point f - the sigmoid notch point of the mandible

**Fig. 4:**
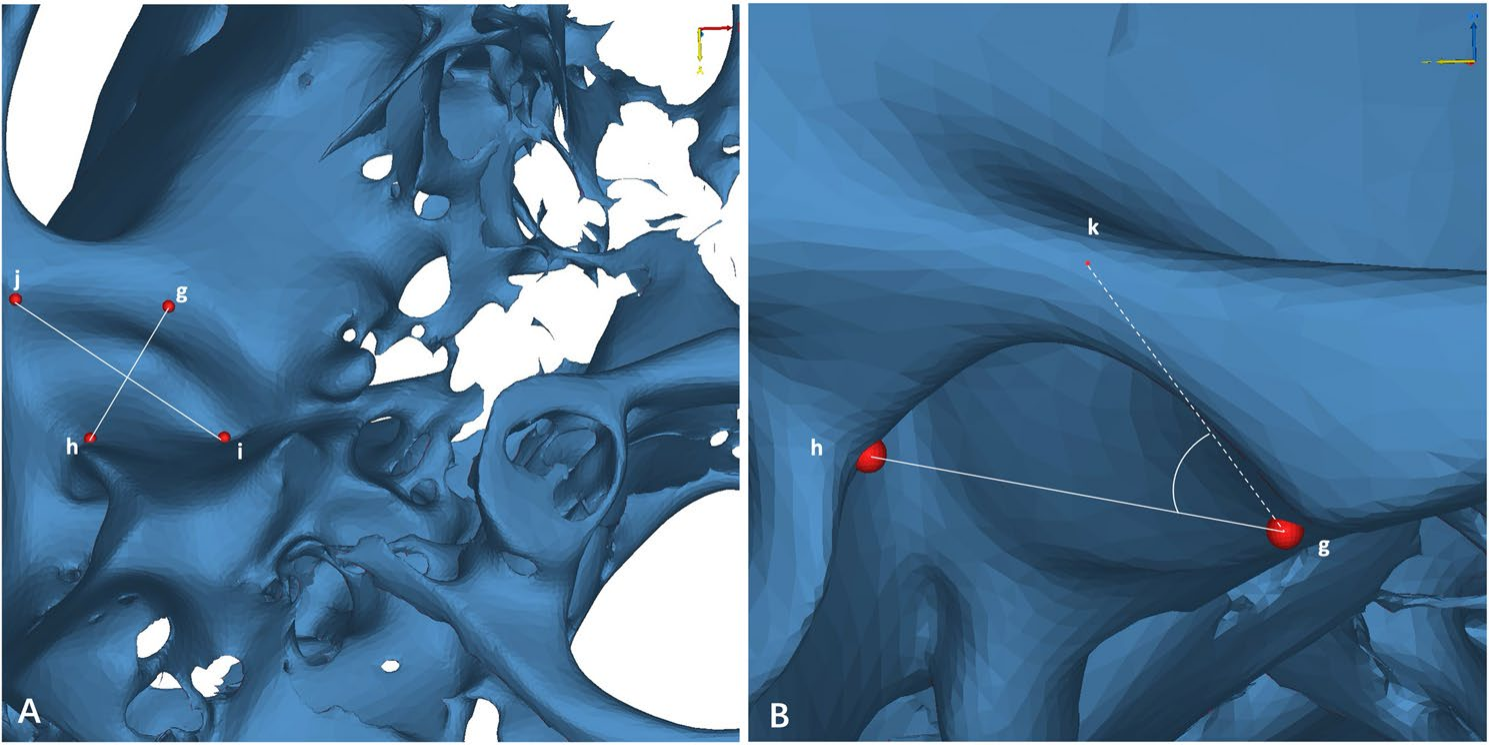
Measurements of the glenoid fossa. **A** line gh -the distance between the anterior and posterior points of the glenoid fossa, line ij -the distance between the medial and lateral points of the glenoid fossa; **B** angle AEI-the angle formed by the apex(k), anterior(g), and posterior(h) points of the glenoid fossa

**Fig. 5:**
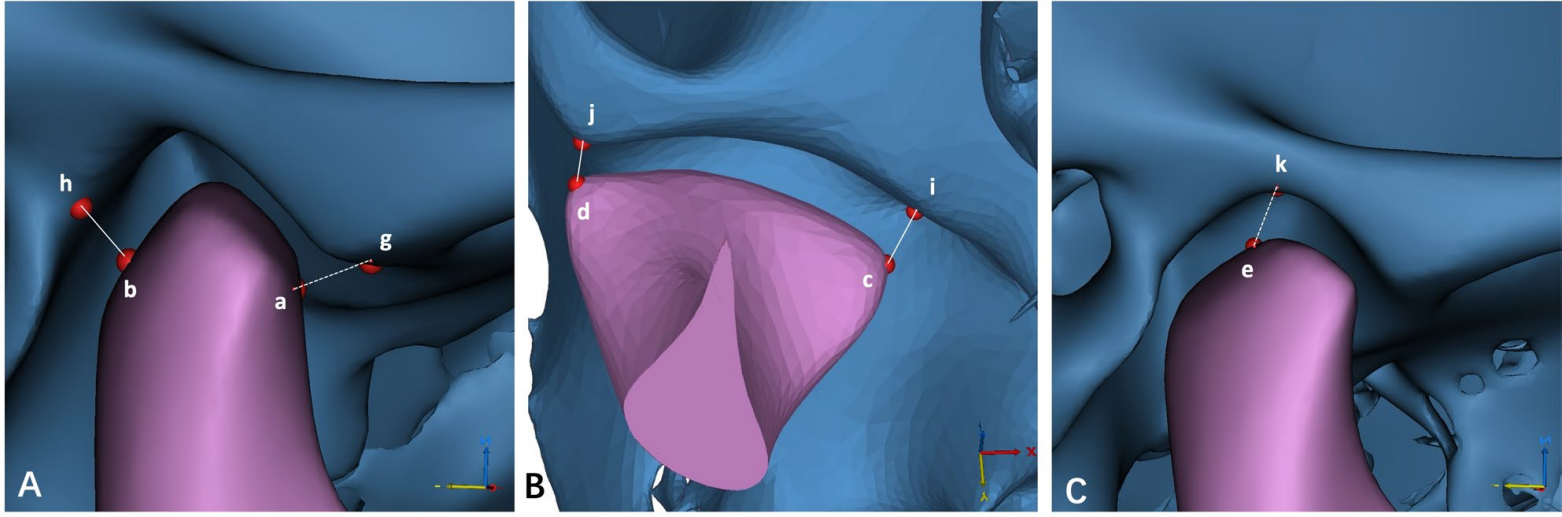
Measurements of TMJ spaces. **A** line ag - anterior joint space/the distance between the anterior point of the condyle and the anterior point of the glenoid fossa, line bh - posterior joint space/the distance between the posterior point of the condyle and the posterior point of the glenoid fossa; **B **line ci - medial joint space/the distance between the medial point of the condyle and the medial point of the glenoid fossa, line dj - lateral joint space/the distance between the lateral point of the condyle and the lateral point of the glenoid fossa; **C** line ek - superior joint space/the distance between the apex point of the condyle and the apex point of the glenoid fossa

Before measurement, each 3D model was oriented using three standard reference planes: the FH plane, the midsagittal plane, and the coronal plane (Table 1; Fig. 2). Anatomical landmarks of the condyle and glenoid fossa were then identified (Table 1), and all measurement definitions were shown in Table 2; Figs. 3, 4 and 5.

**Table 1:** Definition of landmarks and planes

Landmarks / Planes	Definition
Skeletal landmarks	
Nasion (Na)	The highest and most forward point of the frontonasal suture.
Sella (S)	The central point of the hypophyseal fossa located in the middle cranial fossa.
Orbitale (Or)	The most inferior point on the lower margin of the orbit.
Porion (Po)	The most lateral and superior point of the bony margin of the external auditory Meatus.
Reference Planes	
FH plane	A plane passing through right Or, left Or and right Po.
Midsagittal plane	A plane perpendicular to the FH plane and passing through Na and the midpoint of bilateral S.
Coronal plane	A plane perpendicular to the FH plane and the midsagittal plane passing through S.
Condyle landmarks	
Anterior Point	The most anterior point on the anterior slope of the condylar head.
Posterior Point	The most posterior point on the posterior slope of the condylar head.
Medial Point	The most medial point on the condylar head.
Lateral Point	The most lateral point on the condylar head.
Apex Point	The highest point on the condylar head.
Sigmoid notch point	The lowest point of the sigmoid notch.
Glenoid Fossa landmarks	
Anterior Point	The most anterior point of the glenoid fossa.
Posterior Point	The most posterior point of the glenoid fossa.
Medial Point	The most medial point of the glenoid fossa.
Lateral Point	The most lateral point of the glenoid fossa.
Apex Point	The highest point of the glenoid fossa.

**Table 2:** Measurements definition of the Condyle, the glenoid fossa and the TMJ space

Measurements	Definition
Condyle (C)	
Anteroposterior Diameter of Condyle (AP-C) (mm)	The distance between the anterior and posterior points of the condyle.
Mediolateral Diameter of Condyle (ML-C) (mm)	The distance between the medial and lateral points of the condyle.
Surface Area of Condyle (S-C) (mm^2^) and Volume of Condyle (V-C) (mm^3^)	The area and volume calculated by sectioning the mandibular 3D model from the plane parallel to the FH plane and passing through the sigmoid notch point.
Glenoid Fossa (GF)	
Anteroposterior Diameter of Glenoid Fossa (AP-GF) (mm)	The distance between the anterior and posterior points of the glenoid fossa.
Mediolateral Diameter of Glenoid Fossa (ML-GF) (mm)	The distance between the medial and lateral points of the glenoid fossa.
Articular Eminence Inclination (AEI) (°)	The angle formed by the apex, anterior, and posterior points of the glenoid fossa.
Joint Space (JS)	
Anterior Joint Space (A-JS) (mm)	The distance between the anterior point of the condyle and the anterior point of the glenoid fossa.
Posterior Joint Space (P-JS) (mm)	The distance between the posterior point of the condyle and the posterior point of the glenoid fossa.
Medial Joint Space (M-JS) (mm)	The distance between the medial point of the condyle and the medial point of the glenoid fossa.
Lateral Joint Space (L-JS) (mm)	The distance between the lateral point of the condyle and the lateral point of the glenoid fossa.
Superior Joint Space (S-JS) (mm)	The distance between the apex point of the condyle and the apex point of the glenoid fossa.

All reconstructions, landmark identifications, and all measurements were completed in a single session by one examiner with five years of clinical experience and specialized training in TMJ morphology to ensure methodological consistency.

### Statistical methods

Sample size estimation was performed using G*Power software (version 3.1; Universität Kiel). For the one-way Analysis of Variance (ANOVA), based on relevant literature [6, 12], a total sample size of 66 was required (α = 0.05, power = 0.80, effect size f = 0.4), corresponding to at least 22 subjects per group. For the paired t-test, the minimum required sample size was 15 matched pairs (α = 0.05, power = 0.80, effect size d = 0.8).

Data analyses were conducted using SPSS (version 27.0) and GraphPad Prism (version 10.2). Normality was assessed using the Shapiro–Wilk test (*n* < 50) or Kolmogorov–Smirnov test (*n* > 50), and homogeneity of variance using Bartlett’s test. Gender distribution was compared with the Chi-square test. For age and TMJ measurements meeting normality and homoscedasticity, one-way ANOVA with Tukey’s post hoc test was applied; otherwise, Welch’s test with Dunnett’s T3 multiple comparisons was used. Non-normally distributed data were analyzed using the Kruskal–Wallis test with Dunn’s multiple comparisons.

The effects of duration of tooth loss, first molar loss, and UCH were evaluated by comparing the missing side with the contralateral side. Paired t-tests were applied for normally distributed variables, and Wilcoxon signed-rank tests for non-normal data. Statistical significance was set at *P* < 0.05.

Measurement reliability was assessed in 30 randomly selected samples remeasured one month later. Intraclass correlation coefficients (ICC) exceeded 0.95, indicating excellent reliability.

## Results

### Baseline characteristics of study subjects

Based on the inclusion and exclusion criteria, subjects were divided into three groups (Fig. 1): the control group (68 joint sides), the missing group (34 joint sides), and the contralateral group (34 joint sides). Gender distribution did not differ significantly among the groups, whereas age showed significant variation, with the missing and contralateral groups being older (Table 3).

**Table 3:** Gender and age distribution across control, missing, and contralateral groups

Group	Sex		*P*	Age		*P*
	Male(n)	Female(n)		Mean ± SD	Median (Q1, Q3)	
Control	24	44	0.936	27.26 ± 5.63	27 (23, 30)	< 0.0001
Missing	11	23		37.32 ± 13.53	32.5 (27.75, 49.25)	
Contralateral	11	23		37.32 ± 13.53	32.5 (27.75, 49.25)	

Gender distribution was compared with the Chi-square test; Age distribution was compared with the non-parametric test

Because participants with UPTL had a higher median age than controls, we further stratified them into < 33 and ≥ 33 years to evaluate potential age-related effects. No significant differences were found on either the missing or contralateral side (all *P* > 0.05; Appendix Table 1).

Paired t-tests also showed no significant bilateral differences in condyle, glenoid fossa, or joint space measurements in the control group (all *P*-values > 0.05; Appendix Table 2), indicating essentially bilateral symmetry in individuals with intact dentition.

### Comparison of the TMJ structures in three groups

**Fig. 6:**
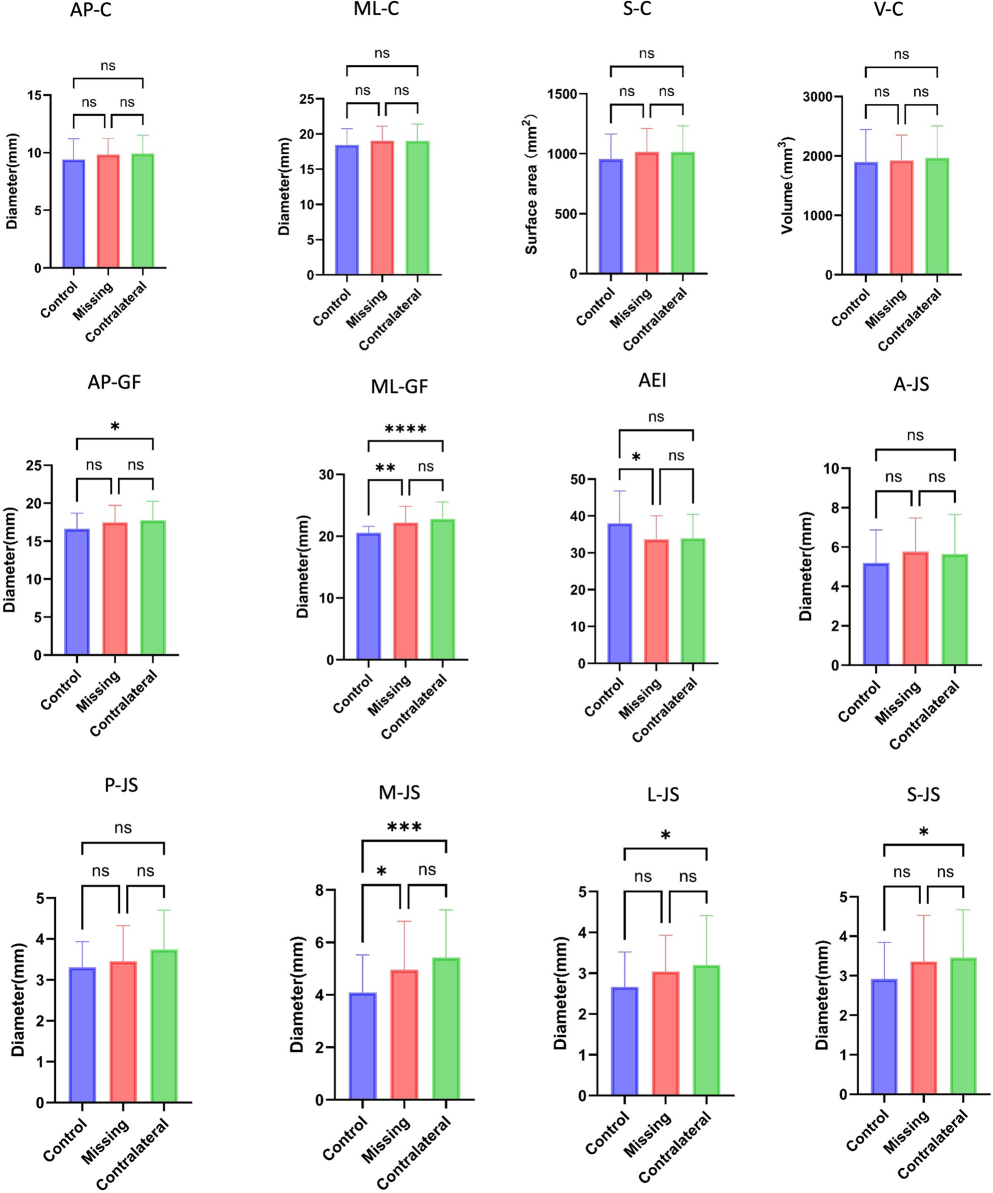
Comparative bar chart analysis of the TMJ structures in control, missing, and contralateral groups. Data were analyzed using ANOVA with Tukey’s test or Kruskal–Wallis with Dunn’s test; p-values were adjusted for multiple comparisons. *-*p* <0.05 **-*p* <0.01 ***-*p* <0.001 ****-*p* <0.0001 ns- *p*≥0.05

A comparative analysis of TMJ structures was conducted among the control, missing, and contralateral groups. As shown in Table 4; Fig. 6, significant differences were observed among the three groups in AP-GF, ML-GF, AEI, M-JS, L-JS, and S-JS (*P* < 0.05).

**Table 4:** Descriptive and comparative analysis of the TMJ in control, missing, and contralateral groups

Variable	Control (*n* = 68)		Missing (*n* = 34)		Contralateral (*n* = 34)		*P*
	Mean ± SD	Median (Q1, Q3)	Mean ± SD	Median (Q1, Q3)	Mean ± SD	Median (Q1, Q3)	
AP-C (mm)	9.418 ± 1.785	9.351 (8.224, 10.21)	9.831 ± 1.401	9.976 (9.023, 11.02)	9.920 ± 1.588	9.857 (8.681, 11.21)	0.2660
ML-C (mm)	18.46 ± 2.286	18.86 (17.23, 19.85)	19.04 ± 2.039	19.16 (17.58, 20.51)	19.01 ± 2.386	19.49 (17.31, 20.61)	0.3361
S-C (mm^2^)	956.1 ± 206.9	961.2 (822.4, 1041)	1014 ± 198.6	993.7 (899.4, 1074)	1014 ± 219.4	1024 (861.6, 1160)	0.1347#
V-C (mm^3^)	1896 ± 549.7	1812 (1464, 2144)	1925 ± 426.6	1895 (1632, 2216)	1967 ± 536.2	2021 (1590, 2427)	0.3588#
AP-GF (mm)	16.65 ± 2.042 b	16.72 (15.27, 18.05)	17.48 ± 2.242 ab	17.38 (16.01, 19.66)	17.75 ± 2.503 a	17.42 (16.40, 19.16)	0.0374 (< 0.05)*
ML-GF (mm)	20.57 ± 1.015	20.47 (20.02, 21.35) b	22.18 ± 2.631	21.92 (20.43, 23.15) a	22.78 ± 2.770	22.47 (20.57, 24.42) a	< 0.0001#****
AEI (°)	38.01 ± 8.803	37.59 (31.25, 43.04) a	33.63 ± 6.409	33.02 (29.46, 37.63) b	33.94 ± 6.502	34.70 (31.96, 38.73) ab	0.0193# (< 0.05)*
A-JS (mm)	5.193 ± 1.674	4.921 (4.107, 6.050)	5.761 ± 1.720	5.825 (4.672, 6.798)	5.646 ± 2.006	5.550 (4.072, 6.885)	0.1393#
P-JS (mm)	3.312 ± 0.6247	3.362 (2.816, 3.817)	3.455 ± 0.8724	3.513 (2.896, 3.961)	3.743 ± 0.9621	3.674 (3.045, 4.168)	0.1649#
M-JS (mm)	4.098 ± 1.423 b	3.798 (3.190, 4.959)	4.954 ± 1.849 a	4.577 (3.360, 6.210)	5.424 ± 1.822 a	5.038 (4.267, 6.503)	0.0004 (< 0.001)***
L-JS (mm)	2.677 ± 0.8535	2.489 (2.095, 2.976) b	3.044 ± 0.8825	2.963 (2.439, 3.624) ab	3.203 ± 1.209	3.116 (2.527, 3.566) a	0.0071# (< 0.01)**
S-JS (mm)	2.920 ± 0.9228 b	2.955 (2.220, 3.411)	3.362 ± 1.167 ab	3.355 (2.373, 4.134)	3.460 ± 1.209 a	3.472 (2.604, 4.235)	0.0274 (< 0.05)*

Data were analyzed using one-way ANOVA with Tukey’s post hoc test, or the Kruskal–Wallis test# with Dunn’s multiple comparisons test for non-normally distributed data. Groups sharing the same letter (a, b) indicate no significant difference

*-*p* < 0.05

**-*p* < 0.01

***-*p* < 0.001

****-*p* < 0.0001

### Comparison of the glenoid fossa of TMJ

The AP-GF was markedly larger in the contralateral group than in the control group, while neither group differed significantly from the missing group. Likewise, both the contralateral and missing groups showed greater ML-GF than the control group, with the most pronounced contrast observed between the contralateral and control groups (*P* < 0.0001). Additionally, the AEI was apparently smaller in the missing group compared with the control group (*P* < 0.05), while no significant variation was found between the missing and contralateral groups (Table 4; Fig. 6).

### Comparison of TMJ spaces

The contralateral group exhibited significantly larger M-JS, L-JS, and S-JS compared with the control group (all *P* < 0.05; Table 4; Fig. 6). Among these parameters, only M-JS was greater in the missing group than in the control group (*P* < 0.05). No notable differences were detected between the contralateral and missing groups across all joint space measurements.

### The impact of tooth loss duration on TMJ structural asymmetry

**Fig. 7:**
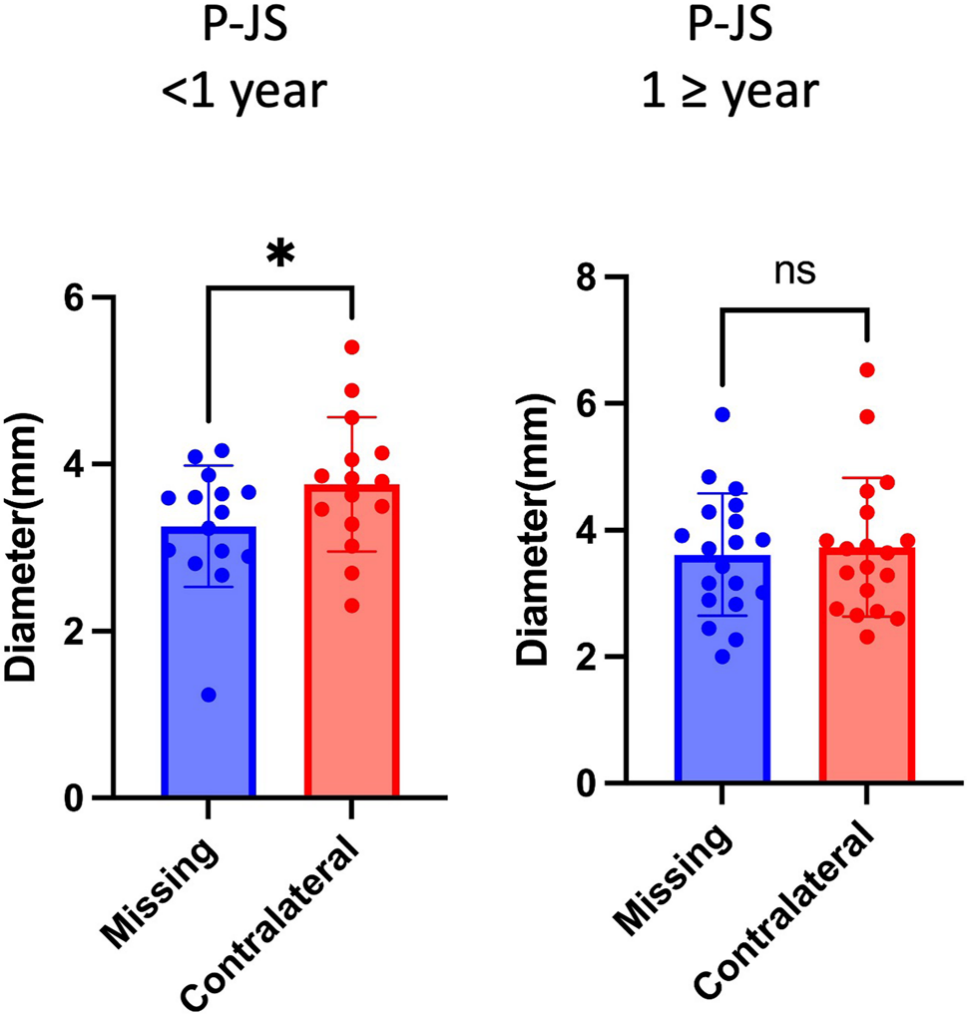
Partial comparative bar chart analysis of TMJ structural asymmetry by tooth loss duration in participants with UPTL. *- *p* <0.05 ns - *p*≥0.05

Participants in the missing group were divided into two subgroups based on the duration of UPTL: <1 year (*n* = 15) and ≥ 1 year (*n* = 19). Within each subgroup, TMJ structures on the missing side were compared with those on the contralateral side. In the subgroup with tooth loss of less than 1 year, the P-JS was significantly smaller on the missing side than on the contralateral side (*P* < 0.05; Table 5; Fig. 7). In contrast, no statistically significant differences in the P-JS were noted between the two sides after more than one year of tooth loss (Table 5; Fig. 7).

**Table 5:** Comparative analysis of TMJ structural asymmetry by tooth loss duration, first molar loss, and UCH in participants with UPTL

Variable	Tooth Loss Duration		*P1*	First Molar Loss	*P2*	UCH	*P*3
	**Year**	Median (Q1, Q3)		Median (Q1, Q3)		Median (Q1, Q3)	
AP-C (mm)	< 1	0.2658 (-0.7057, 0.9960)	0.5553	-0.3654 (-1.100, 0.9170)	0.3779	-1.093 (-1.553, -0.3492)	0.009 (< 0.01)**
	≥ 1	-0.4559 (-1.259, 1.449)	0.4672				
ML-C (mm)	< 1	0.364 (-1.582, 1.273)	0.6271	0.1218 (-1.559, 1.091)	0.9921	-0.364 (-1.372, 1.245)	0.4438
	≥ 1	0.1218 (-1.400, 1.091)	0.7259				
S-C (mm^2^)	< 1	18.21 (-120.7, 194.4)	0.6293	-15.71 (-112.5, 105.6)	0.759	-8.23 (-141.9, 81.12)	0.571
	≥ 1	-15.71 (-102.6, 32.80)	0.4180##				
V-C (mm^3^)	< 1	-0.65 (-385.4, 314.9)	0.7235	-62.31 (-385.4, 141.6)	0.3532	64.36 (-204.7, 277.5)	0.9919
	≥ 1	-12.79 (-294.9, 134.9)	0.1843				
AP-GF (mm)	< 1	-0.7533 (-1.396, 0.5438)	0.0768	-1.056 (-1.636, 0.3058)	0.004 (< 0.01)**	-0.4438 (-1.221, 0.8499)	0.8216
	≥ 1	0.3058 (-1.229, 1.045)	0.7149				
ML-GF (mm)	< 1	0.1648 (-1.318, 1.387)	0.8846	-0.8289 (-2.359, 0.6741)	0.0394 (< 0.05)*	0.6741 (-1.234, 2.810)	0.173
	≥ 1	-0.9498 (-2.564, -0.2219)	0.0589				
AEI (°)	< 1	1.206 (-2.590, 7.509)	0.1917	-1.161 (-5.591, 6.563)	0.9594	1.206 (-6.682, 3.214)	0.4237
	≥ 1	-3.658 (-7.010, 2.6040)	0.1222				
A-JS (mm)	< 1	-0.4597 (-1.014, 1.117)	0.6387##	-0.4597 (-1.014, 0.5935)	0.6869##	-0.5805 (-2.108, 0.9296)	0.4182
	≥ 1	0.4738 (-0.9659, 1.057)	0.5762				
P-JS (mm)	< 1	-0.6299 (-0.9615, -0.2244)	0.038 (< 0.05)*	-0.6299 (-1.113, 0.4335)	0.0656	0.5867 (-0.4840, 0.9043)	0.3151
	≥ 1	0.1726 (-0.8408, 0.5867)	0.6625				
M-JS (mm)	< 1	-0.2352 (-1.548, 0.3802)	0.0888	-0.4964 ( -1.630, 0.6032)	0.2467##	0.2352 (-0.8462, 1.142)	0.8926##
	≥ 1	-0.3667 (-1.066, 0.8021)	0.6507##				
L-JS (mm)	< 1	-0.1193 (-0.8077, 0.4690)	0.5515	-0.1374 (-0.8343, 0.5792)	0.6097	0.1374 (-0.4088, 0.4555)	0.5879##
	≥ 1	-0.06909 (-0.9037, 0.5981)	0.7177				
S-JS (mm)	< 1	-0.3395 (-0.5457, 0.3422)	0.4212##	-0.07092 (-0.7021, 0.3147)	0.3294##	0.3147 (-0.6792, 0.9111)	0.974
	≥ 1	0.11 (-0.8684, 0.4240)	0.383				

Paired t-tests were conducted for normally distributed variables, while the Wilcoxon test## was used for non-normally distributed data. *p*1: Missing vs. contralateral sides by tooth loss duration;*p*2: Missing vs. contralateral sides in first molar loss༛*p*3: Preferred vs. non-preferred chewing sides in UPTL

*-*p* < 0.05

**-*p* < 0.01

### The impact of first molar loss on TMJ structural asymmetry

**Fig. 8:**
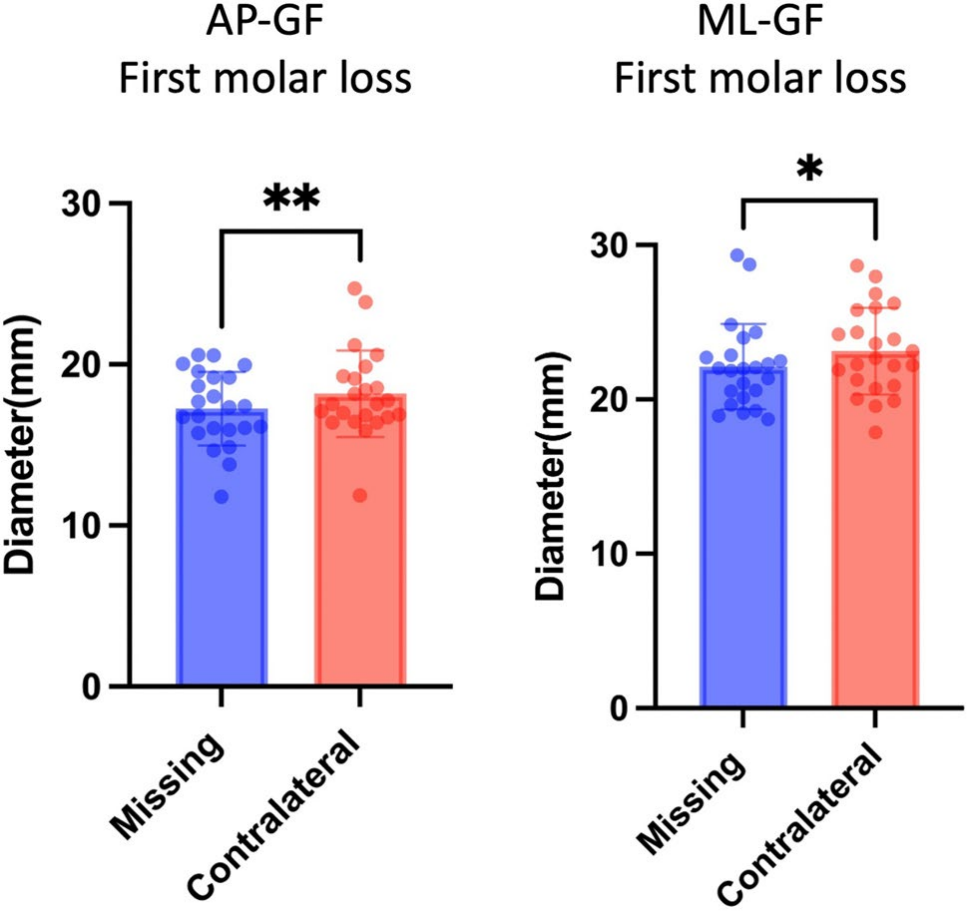
Partial comparative bar chart analysis of TMJ structural asymmetry by first molar loss in participants with UPTL *-*p* <0.05 **-*p* <0.01 ns- *p*≥0.05

In the 23 participants with first molar loss, the glenoid fossa exhibited significant enlargement in both its AP-GF and ML-GF on the contralateral side compared with the missing side (*P* < 0.05; Table 5; Fig. 8).

### The impact of UCH on TMJ structural asymmetry

Among the 34 participants with UPTL, 13 exhibited a UCH, with the contralateral side as the preferred chewing side. The AP-C was significantly reduced on the preferred side than on the non-preferred side (*P* < 0.01; Table 5), suggesting a link between UCH and ipsilateral condylar resorption.

EMR identified 13 participants with UCH. This smaller-than-expected sample size resulted in a statistical power of 0.75 for the paired t-test analysis.

## Discussion

This study comprehensively compared TMJ structures among the missing group, the contralateral group, and the control group, with a particular focus on bilateral comparisons in patients with UPTL rather than simply comparing those with and without tooth loss. In patients with UPTL, the glenoid fossa exhibited increased AP-GF and ML-GF relative to controls, accompanied by corresponding enlargement of M-JS, L-JS, and S-JS. Although the AEI in the missing group was lower than that in the contralateral and control groups, only its difference from the control group reached statistical significance (Table 4; Fig. 6).

Previous research [8, 9, 11, 13, 14] has suggested that tooth loss may reduce the AEI resulting from uneven biomechanical loading, which disrupts mandibular dynamics and promotes bone resorption at the articular eminence. However, these investigations mainly involved edentulous patients [9, 14] or relied on panoramic or cephalometric imaging [11, 13]. The present work confirmed that UPTL induced compensatory remodeling in the glenoid fossa, joint spaces, and AEI, with relatively limited bilateral TMJ differences. Adaptation of the glenoid fossa and joint spaces was more evident on the contralateral side, whereas AEI reduction was most pronounced on the missing side. In contrast, Chen M et al. [15] observed a marked compensatory reduction in contralateral AEI in older patients with unilateral free loss of posterior teeth based on CBCT analysis, likely reflecting prolonged tooth loss and sustained mechanical loading of the articular eminence.

The first permanent molar plays a key role in occlusion [16] and TMJ development, particularly influencing the articular eminence of the glenoid fossa [17, 18]. Its loss can alter occlusal forces, bone metabolism, and joint morphology [19–21]. Among all teeth, the first molar exhibits the highest incidence of loss [22]. Tanaka E et al. [23] demonstrated that posterior tooth loss may overload the TMJ, inducing morphological alterations in its bony structures. In alignment with this mechanism, the present study further revealed that unilateral first molar loss is associated with greater compensatory adaptation of the contralateral glenoid fossa. Although earlier studies [11, 15, 24] also noted flattening of the glenoid fossa in patients with missing teeth, they primarily measured the AEI or fossa depth and did not evaluate AP-GF or ML-GF dimensions. Moreover, they did not specifically address the impact of first molar loss on glenoid fossa symmetry.

Regarding joint spaces, many studies [7, 9, 25, 26] have reported that following tooth loss, A-JP tends to increase while P-JS decreases. However, many of these investigations involved edentulous patients [7, 9] or adolescents [26], and none provided left–right comparisons within the same individuals. Joint space analysis in this study indicated progressive enlargement of M-JS, L-JS, and S-JS from controls to missing sides and further to contralateral sides, filling a research gap regarding M-JS and L-JS. Additionally, Chandran Kana et al. [27] proposed that enlargement of the S-JS may be attributed to bone resorption at the glenoid fossa roof induced by modified mechanical stress on the TMJ after tooth loss. Taken together, these findings suggest that occlusal factors, shifts in condylar position, condylar resorption, and resorptive changes in the glenoid fossa roof may jointly contribute to variations in joint space. These observations underscore the need for further investigation.

Some studies [13, 28, 29] have examined the effects of tooth loss duration on AEI and TMD symptoms, but its impact on joint spaces has rarely been reported. In the present study, tooth loss duration primarily affected P-JS: patients with less than one year of UPTL presented a larger contralateral P-JS compared with the missing side, whereas no differences were identified after more than one year, likely due to accelerated remodeling of the missing side.

Regarding condylar morphology, prior studies [6, 30] have associated tooth loss with condylar flattening, erosion, and reduced diameter. Some authors have suggested that the condylar resorption grade may be related to the number of missing posterior teeth, the number of quadrants affected, and bilateral posterior teeth loss [4]. In the current sample, condylar size did not differ among groups, potentially because participants had relatively few missing teeth. Similarly, a previous cohort study [10] reported no significant association between condylar alterations and molar loss.

Posterior tooth loss often shifts chewing dominance to the contralateral side [31, 32]. Previous studies [32, 33] have primarily focused on the effects of UCH on TMD symptoms. Other investigations [31, 34], which employed methods different from those used in this study to measure condylar size, reported no significant influence of UCH on condylar volume or dimensions. In the present study, patients with UPTL and UCH showed that the contralateral condyle (preferred chewing side) exhibited a smaller AP-C than the missing side. Similarly, Zhai X et al. [35] reported decreased AP-C on the preferred chewing side in patients with UCH, although their sample mainly included individuals with degenerative TMD. Collectively, these observations suggest that UCH may contribute more substantially to condylar resorption than tooth loss itself, particularly in terms of AP-C.

The current work has several limitations. As a retrospective investigation, clinical information such as the duration of tooth loss may be prone to recall bias. The tooth-loss patterns in our sample were relatively homogeneous, as factors such as free loss of posterior teeth or the number of missing teeth were not further stratified. Furthermore, measurements of condylar position and glenoid fossa roof thickness were not included, both of which may be relevant to changes in joint space.

## Conclusions

This study demonstrated that UPTL can induce morphological alterations in the TMJ, with the glenoid fossa showing more susceptibility to bone resorption than the condyle. Through a refined grouping strategy and comprehensive CBCT assessment (including condylar dimensions, glenoid fossa size and morphology, and joint space dimensions), this study further identified several patterns of structural adaptation. First molar loss was associated with compensatory enlargement of the contralateral glenoid fossa, while UCH appeared to contribute more strongly to condylar resorption on the preferred chewing side than tooth loss alone. Furthermore, an early compensatory increase in contralateral P-JS was observed, followed by later adaptation on the missing side. These findings provide new insight into the bilateral differences in the dynamic remodeling of the TMJ following UPTL.

These findings highlight the clinical importance of early restoration of posterior teeth, particularly the first molar, to minimize compensatory TMJ remodeling and reduce the risk of joint asymmetry. Clinicians should carefully evaluate the presence of UCH during treatment planning, as UCH may exacerbate condylar degeneration and compromise long-term joint stability. Consideration of functional habits alongside occlusal rehabilitation may therefore be essential for preserving TMJ structure and function.

Future studies should incorporate both the signs and symptoms of TMD and perform stratified analyses based on factors such as age, sex, and tooth-loss patterns. In addition, research should not be limited to comprehensive CBCT measurements of bony structures, but also include Magnetic Resonance Imaging (MRI) assessments of soft tissues, such as the articular disc [36]. These approaches will help to further validate the effects of various tooth loss conditions on TMJ morphology and TMD symptoms.

## Supplementary Information

Supplementary Material 1.

Supplementary Material 2.

## Data Availability

The datasets used and/or analyzed during the current study are available from the corresponding author on reasonable request.

## Abbreviations

UPTL: Unilateral posterior tooth loss
TMJ: Temporomandibular joint
CBCT: Cone-beam computed tomography
3D: Three-dimensional
UCH: Unilateral chewing habit
2D: Two-dimensional
EMR: Electronic medical records
TMD: Temporomandibular Disorders
DICOM: Digital Imaging and Communications in Medicine
STL: Stereolithography
FH plane: Frankfort Horizontal plane
Na: Nasion
S: Sella
Or: Orbitale
Po: Porion
C: Condyle
AP-C: Anteroposterior Diameter of Condyle
ML-C: Mediolateral Diameter of Condyle
S-C: Surface Area of Condyle
V-C: Volume of Condyle
AP-GF: Anteroposterior Diameter of Glenoid Fossa
ML-GF: Mediolateral Diameter of Glenoid Fossa
AEI: Articular Eminence Inclination
JS: Joint Space
A-JS: Anterior Joint Space
P-JS: Posterior Joint Space
M-JS: Medial Joint Space
L-JS: Lateral Joint Space
S-JS: Superior Joint Space
ANOVA: Analysis of Variance
ICC: Intraclass correlation coefficients
MRI: Magnetic Resonance Imaging
